# Increased expression of the leptin receptor in human ovaries affected by endometrioma and detection of high levels of leptin in the ovarian endometriomal fluid

**DOI:** 10.1186/1757-2215-7-2

**Published:** 2014-01-08

**Authors:** Carolina Zendron, Helder F Gonçalves, Fernanda S Cavalcante, Thiago RD Pereira, Alessandra Evangelista, Cristiane F Ramos, Marco Aurelio P Oliveira

**Affiliations:** 1 Department of Gynecology, Rio de Janeiro State University, 20551-030, Rio de Janeiro Brazil; 2 Laboratory of Morphometry, Metabolism and Cardiovascular Diseases, Department of Anatomy, Rio de Janeiro State University, 20551-030, Rio de Janeiro Brazil

**Keywords:** Leptin, Leptin receptor, Ovary, Ovarian endometrioma, Endometriomal fluid endometriosis

## Abstract

**Background:**

This study was designed to investigate leptin levels in the fluid in ovarian endometriomas (OEs) and to compare the expression of leptin and its receptors (OBR) in ovarian tissue affected by endometrioma in infertile women to its expression in the normal ovarian tissue of fertile controls without endometriosis.

**Methods:**

In this case–control observational study, ovarian tissue, blood samples and peritoneal fluid were obtained from 20 women (10 fertile controls without endometriosis or any ovarian disease, who were undergoing tubal ligation surgery, and 10 infertile women with severe endometriosis and OE). The ovarian endometriomal fluid (EF) was aspirated, and peritoneal-implant (PI) biopsies were performed. The tissues removed during the surgeries were immediately frozen in liquid nitrogen to determine expression levels by western blot and leptin levels by ELISA.

**Results:**

OBR was expressed at higher levels in the ovarian tissue affected by endometrioma than in the normal ovarian tissue (control = 0.38 ± 0.05, study = 0.60 ± 0.09, p = 0.03), but there was no significant difference in leptin levels between these groups (control = 0.57 ± 0.1, study = 0.35 ± 0.1, p = 0.18). Positive and significant correlations were observed between leptin and OBR in the OE (r = 0.85, p = 0.004) and in the PI (r = 0.87, p = 0.001). ELISA results demonstrate a greater leptin concentration within the EF compared with the serum and the PF (serum = 14.25 ± 1.63, PF = 5.98 ± 2.0, EF = 73.8 ± 16.2, p = 0.0001), but there was no correlation between these variables. A positive, significant and strong correlation was observed between PF leptin levels and the expression of leptin and OBR in PI (leptin: r = 0.78, p = 0.007/OBR: r = 0.68, p = 0.04) and between the EF leptin levels and the expression of leptin and OBR in the OE (leptin: r = 0.88, p = 0.008/OBR: r = 0.89, p = 0.005).

**Conclusions:**

These data suggest that leptin may play an important role in the physiopathology of OE through a modulatory interaction with its active receptor.

## Background

Leptin, the product of the ob/ob gene [1] is an adipocyte-derived protein that regulates food intake and energy expenditure. Accumulating evidence shows that it is also a crucial factor in the endocrine regulation of several physiologic processes, including inflammation, angiogenesis and reproductive functions [2].

Endometriosis is a chronic and progressive disease associated with abnormal peritoneal and endometrial production of proinflammatory cytokines, growth factors and angiogenic factors [3], which may interfere with the function of the reproductive system.

Due to its inflammatory and angiogenic properties, as well as its possible involvement in reproductive abnormalities at both the central and the gonadal levels [4], leptin has been extensively studied in patients with endometriosis. A recent report demonstrated that leptin signaling is a necessary component of lesion proliferation, early vascular recruitment, and the maintenance of neoangiogenesis in a murine model of endometriosis [5]. Another report showed that the leptin receptor (OBR) is induced in endometriosis and that leptin stimulates the growth of endometriotic epithelial cells through the JAK2/STAT3 and ERK pathways [6].

Endometrioma is a localized form of endometriosis that primarily affects the ovaries and occurs in approximately 17-40% of women with endometriosis [7]. The pathogenesis of endometriotic ovarian cysts remains controversial, and their treatment remains a challenge. Ovarian endometriomas (OEs) form through progressive invagination of the ovarian cortex [8], suggesting that they are false cysts and that the cyst wall is made of the same material as the ovarian cortex [9]. OEs equal to or larger than 3 cm respond poorly to medical therapy [10], and both OEs and their surgical removal are associated with a significant reduction in the ovarian reserve, with negative effects on fertility [11].

The expression of leptin and its receptor has been described in OEs [12]. Small studies have demonstrated an increased concentration of this peptide in the peritoneal fluid (PF) of patients with endometriosis [13], and it is present at higher levels in women with peritoneal endometriosis than in women with ovarian endometriosis. Based on these findings, Alvigii suggests that patients with OE may show increased leptin levels in the ‘chocolate’ fluid in the endometrioma [14], but there is insufficient evidence to support this hypothesis.

As suggested by previous studies, leptin has a role in the pathogenesis of OE via inflammatory and angiogenic effects; however, no study had compared the expression of this protein in human ovarian tissue affected by endometrioma to its expression in normal ovarian tissue, and its presence in the chocolate fluid in OEs has never been investigated.

This study was designed to compare the expression of leptin and its receptors in ovarian tissue affected by endometrioma in infertile women to its expression in the normal ovarian tissue of fertile controls not affected by endometriosis. We also examine, for the first time, leptin levels in the ovarian endometriomal fluid (EF).

## Methods

### Patient enrollment

The study group consisted of ten patients who underwent laparotomy or laparoscopy for adnexal masses and infertility (n = 10). The inclusion criteria for this group were at least one year of primary infertility; regular cycles before starting hormonal treatment to control pain associated with endometriosis; unilateral or bilateral OE and normal male fertility. Peritoneal endometriotic lesions were observed in all patients in the study group. The control group was composed of ten women with proven fertility from the family-planning program of the same hospital who were undergoing mini-laparotomy or laparoscopy for tubal ligation and without surgical evidence of endometriosis or any ovarian pathology. All patients in the control group had a normal pelvic cavity. The surgeries were performed between February 8, 2013, and July 31, 2013, at the Department of Gynecology of the Pedro Ernesto University Hospital, Rio de Janeiro.

All of the subjects were of reproductive age and were receiving hormonal therapy for clinical treatment of pain associated with endometriosis or for contraception (in the control group). All enrolled patients had a body mass index (BMI) of 20–30 kg/m^2^. The exclusion criteria were clinical and/or echographic indications of polycystic ovarian disease, diabetes and systemic hepatic or thyroid inflammatory disease and surgical evidence of any other ovarian pathology. The study was approved by the local ethics committee (Research Ethics Committee of the Pedro Ernesto University Hospital (CEP - HUPE), Rio de Janeiro, Brazil), and written informed consent was obtained from all patients before the procedures.

### Tissue specimens

Serum samples were obtained before anesthesia. PF was aspirated from the posterior cul-de-sac at the beginning of surgery. A small wedge resection of the intact and healthy ovary was performed in the control group. The ovarian EF was aspirated, and the OE was removed, always by the same surgeon by cystectomy. Peritoneal biopsies were performed in the study group to provide histological confirmation of endometriosis and data for the study.

The extent of endometriosis was scored according to the revised standards of the American Society of Reproductive Medicine [15]. A portion of each sample was sent to a pathologist, who reviewed the ovarian endometriomal specimens to confirm the presence of cyst wall-lining cells and ovarian-cortex cells, and normal ovary specimens were examined to confirm the absence of pathology. All samples used in the study were immediately frozen in liquid nitrogen and stored at – 80°C.

### Western blotting

Approximately 500 mg of tissue was homogenized in 500 μl of lysis buffer containing 1% NP-40 (Amresco, Ohio, USA) and a protease-inhibitor mix (Sigma), then centrifuged at 9700 rpm at 4°C. The protein concentration was measured by fluorometry (Qubit 2.0, Life Technologies Corporation, CA, USA), and 20-μg aliquots were applied to 8% SDS-polyacrylamide gel and submitted to vertical electrophoresis (150 mA, 50 V for 90 min), then transferred to nitrocellulose membranes (80 mA, 4 V for 90 min) in a semi-dry transfer apparatus. The membranes were subsequently incubated with antibodies (Santa Cruz, CA, USA) to leptin (1:500) and OBR (1:200). The expression of the proteins under study was normalized against the expression of β-actin. The bands were visualized by chemiluminescence (ECL, Amersham Biosciences, Piscataway, NJ, USA), and documented on the ChemiDoc MP System, Bio-Rad (Life Science Research, USA). All bands were quantified using Image J software 1.42q, USA.

### Determining levels of leptin

The concentration (ng/ml) of leptin in serum, PF and EF was determined by ELISA (Millipore Corporation Billerica, MA, USA). The spectrophotometer was read according to the manufacturer’s specifications.

### Statistical analyses

We used GraphPad Prism (version 6.0, GraphPad Software, CA, USA) to test data for normality and homogeneity of variances. Student’s t-test was used to compare the two groups, and analysis of variance was used to compare three groups. Pearson’s correlation was performed to examine the correlations between some parameters. All results are reported as the mean ± standard error of the mean (SEM), and P-values < 0.05 were considered statistically significant.

## Results

The age and BMI of the patients are expressed as the mean ± the standard error (Table 1). All patients were classified as having stage-IV (severe) endometriosis, and in all patients in the study group, surgery was indicated by infertility associated with an adnexal mass. In the control group, all patients underwent surgery for tubal ligation. One inclusion criterion was the use of hormone therapy; 80% of patients in the study group and 60% of patients in the control group were using a combined oral contraceptive (estrogen and progesterone), and the remaining patients in both groups were using isolated progestin therapy.

**Table 1:** Baseline characteristics of participants

	**Control**	**Study**
*Demographic and anthropometric variables*		
N	10	10
Age (yr)	31.5 +/− 1.63	30.5 +/− 1.50
Body mass index (kg/m^2^)	23.4 +/− 0.88	23.6 +/− 0.57
*Stage of disease*		
Stage I (minimal)	-	0 (0)
Stage II (mild)	-	0 (0)
Stage III (moderate)	-	0 (0)
Stage IV (severe)	-	10 (100)
*Hormonal therapy*		
Estrogen + progesterone	6 (60)	8 (80)
Progesterone	4 (40)	2 (20)
*Indication for surgery*		
Infertility + adnexal mass	-	10 (100)
Tubal ligation	10 (100)	-

Values in parentheses are percentages.

Age and BMI are expressed as the mean +/−.

**Figure 1:**
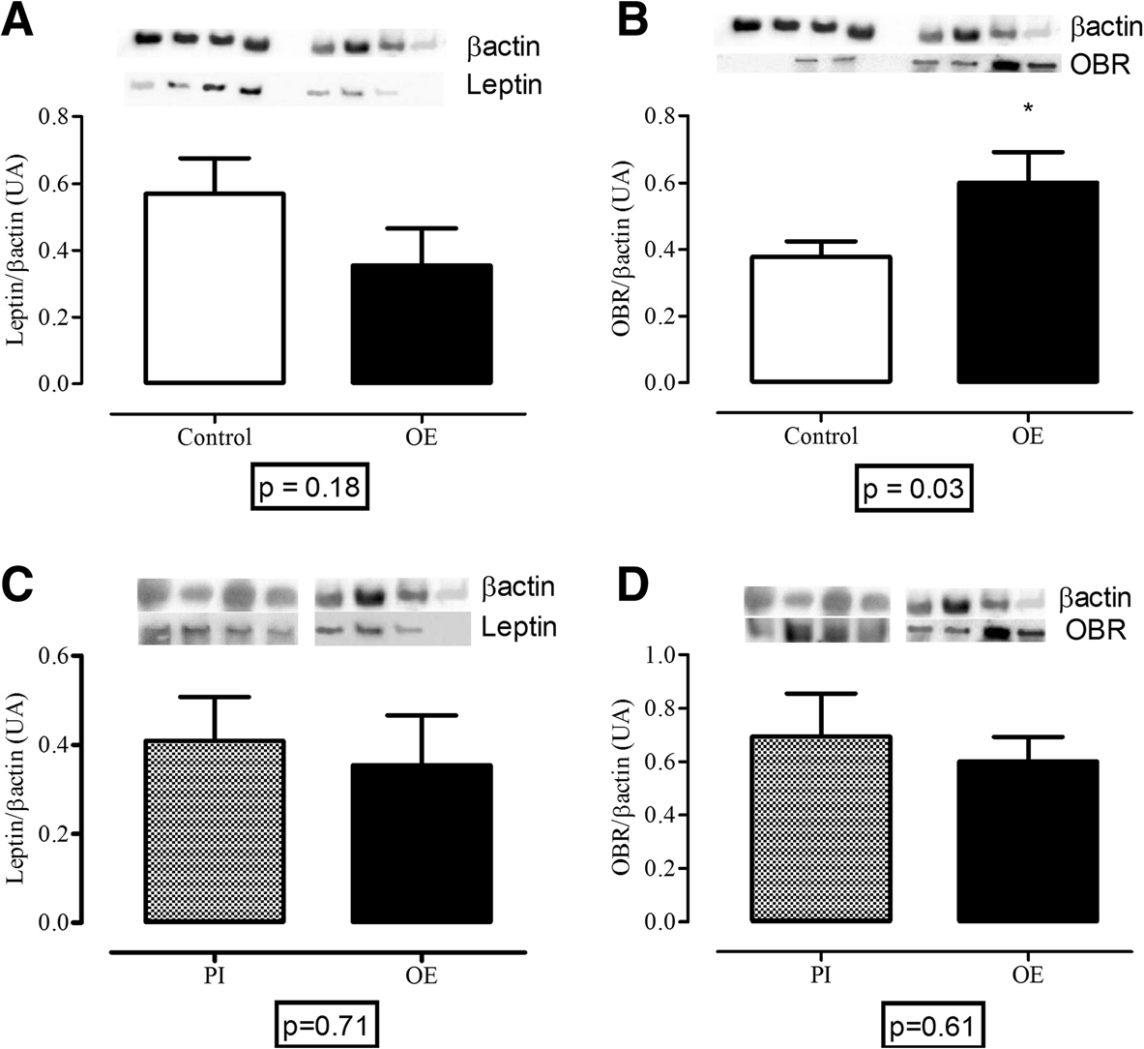
**Expression of leptin and the leptin receptor in the control and ovarian endometrioma groups.** Expression of leptin **(A)** and the leptin receptor (OBR; **B**) in the control and ovarian endometrioma (OE) groups; and expression of leptin **(C)** and the leptin receptor (OBR; **D**) in endometriotic peritoneal implants (PI) and in ovarian endometrioma (OE) in the study group. Data are expressed as the mean +/− SEM per group of 10 subjects.

Western blots revealed no significant decrease in leptin levels in the study group (control = 0.57 ± 0.1, study = 0.35 ± 0.1, p = 0.18), as shown in Figure 1A. In contrast, the receptor (Figure 1B) was expressed at significantly higher levels in the same group (control = 0.38 ± 0.05, study = 0.60 ± 0.09, p = 0.03). In the study group, there was no significant difference in the expression of leptin (PI = 0.40 ± 0.09, OE = 0.35 ± 0.11, p = 0,71) and its receptors (PI = 0.69 ± 0.16, OE = 0.60 ± 0.09, p = 0.61) between patients with ovarian OE and those with peritoneal implants (PI; Figure 1C and D).

**Figure 2:**
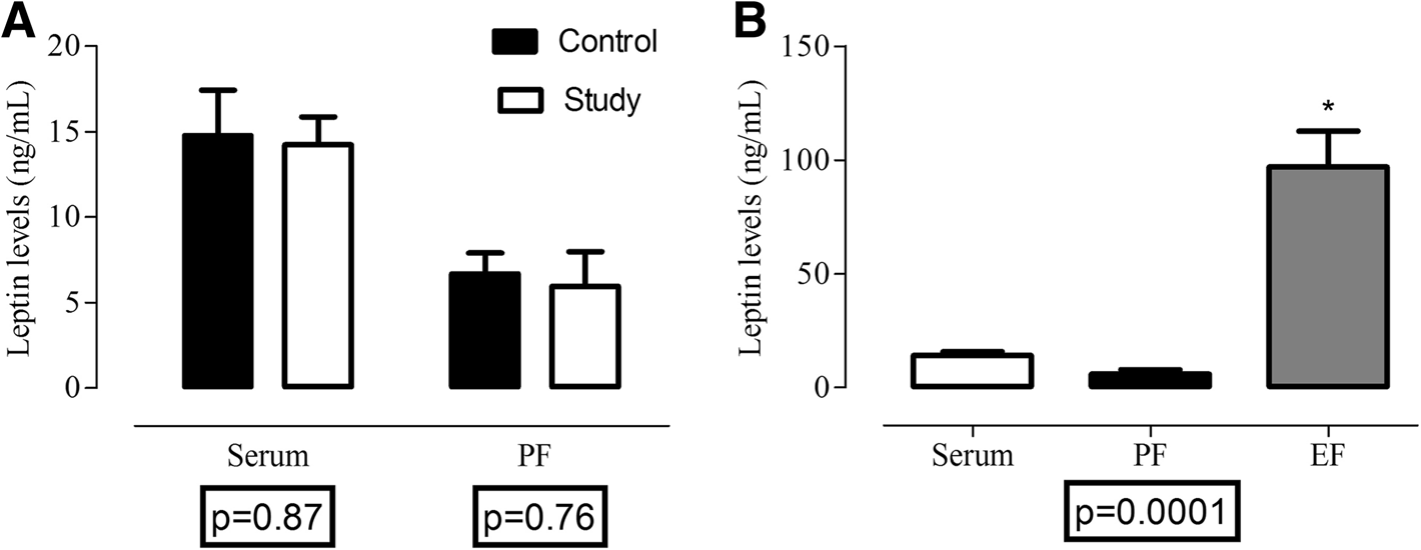
**Leptin levels in the control and study groups.** Leptin levels in serum and the peritoneal fluid (PF) in the control and study groups **(A)**. Leptin levels in the serum, peritoneal fluid (PF) and endometriomal fluid (EF) of patients in the study group **(B)**. Data expressed as the mean +/− SEM per group of 7 subjects.

There was no difference in serum (control = 14.79 ± 2.63, study = 14.25 ± 1.63, p = 0.87) and in PF (control = 6.68 ± 1.21, study = 5.98 ± 2.0, p = 0.76) leptin levels in the control group compared to the study group (Figure 2A). The leptin levels in the serum, PF and EF of patients in the study group are presented in Figure 2B. The leptin levels in the EF were significantly higher than those in the serum and PF (serum = 14.25 ± 1.63, PF = 5.98 ± 2.0, EF = 73.8 ± 16.2, p = 0.0001).

Leptin levels in the serum, PF and EF did not show any correlation between each other, in patients with OE. The correlation between leptin and OBR are presented in Table 2. A positive and significant correlation was observed between leptin and OBR expression in the OE (r = 0.85, p = 0.004) and PI (r = 0.87, p = 0.001) of patients in the study group, but this relationship was not observed in the control group (r = 0.41, p = 0.22). There was no correlation between leptin levels in the PF and the expression of leptin and OBR in the OE (leptin = 0.75, p = 0.08/OBR: r = −0.17, p = 0.71; Table 2), and this correlation was positive and significant for PI (leptin: r = 0.78, p = 0.007/OBR: r = 0.68, p = 0.04). Leptin levels in the EF correlated strongly and positively with the expression of leptin and OBR in the OE (leptin: r = 0.88, p = 0.008/OBR: r = 0.89, p = 0.005; Table 2).

**Table 2:** Correlation among leptin and its receptors in ovarian tissue and peritoneal implants

	**Correlation coefficient**	**P value**
*Ovarian tissue*		
Leptin OE and OBR OE	0.85	0.004
Leptin Control and OBR Control	0.41	0.22
Leptin OE and PF leptin	0.75	0.08
OBR OE and PF leptin	- 0.17	0.71
Leptin OE and EF leptin	0.88	0.008
OBR OE and EF leptin	0.89	0.005
*Peritoneal implants*		
Leptin PI and OBR PI	0.87	0.001
Leptin PI and PF leptin	0.78	0.007
OBR PI and PF leptin	0.68	0.04

Pearson correlation analysis. *OE* ovarian endometrioma, *OBR* leptin receptor, *PF* peritoneal fluid, *EF* endometriomal fluid, *PI* peritoneal implants.

## Discussion

This observational case–control study showed that OBR is expressed at higher levels in ovarian tissue affected by endometrioma in infertile patients than in the normal ovarian tissue of fertile controls not affected by endometriosis. In contrast, leptin expression was slightly lower in the study group. These findings have never previously been described in the literature. Previous studies have used normal endometrium or PI in patients with endometriosis as control groups, whereas we used normal ovarian tissue. Wu et al. detected the leptin transcript and protein in both PI and OE and found no difference in the quantity of leptin transcript between these two groups [12]; however, the expression of leptin and OBR mRNA is increased in OEs compared with the normal endometrium. We also compared the expression of leptin and its receptors in the OE to its expression in PI in patients in our study group; as in the previous study, we found no difference between these two groups. Recently, the expression of leptin and OBR was found to be significantly higher in the OE than in normal endometrium [16]. Moreover, this same report showed that treatment of endometriotic cells with leptin induced the expression of OBR mRNA, which suggests autocrine and paracrine involvement of the leptin system in endometriosis. These data suggest that endometriosis implants are both a potential source of leptin production and a potential target of its action. Therefore, we suggest that ovarian tissue affected by endometrioma might be more responsive to leptin than normal ovarian tissue and might also have a greater capacity for synthesis of this peptide.

Although these groups are small, their relative homogeneity is a strength of this study. All women in the study group had infertility and stage-IV (severe) endometriosis. The stage of endometriosis is not correlated with the presence or severity of symptoms, but infertility is very likely in patients with stage IV endometriosis [15]. All women in the control group were fertile and underwent surgery for tubal ligation. Most studies include different stages of endometriosis and other pelvic diseases, such as uterine leiomyoma or cancer in the controls, introducing potential bias. All women in this study were receiving hormone therapy, which provided a stable hormonal environment and eliminated the possibility of fluctuations in leptin levels during the menstrual cycle.

Our findings demonstrated no difference in PF leptin levels in infertile women with severe endometriosis and OE compared to fertile controls not affected by endometriosis and similar serum leptin levels in both groups. Serum leptin levels appear to be similar in women with and without endometriosis at any stage [17]. In contrast, small studies have shown that PF leptin is significantly higher in endometriosis patients compared to those without the disease and the presence of OE had no significant main effect on leptin concentration. [18]. PF leptin levels were significantly higher in infertile women with endometriosis than in patients with pelvic pain and endometriosis [19] or unexplained infertility [20]. Nevertheless, PF leptin levels were inversely correlated with the stage of disease, which could explain our result [21].

PF leptin levels in patients with OE are elevated due to peritoneal endometriotic lesions or OE; the cause is presently unknown. One report showed that patients with “superficial” endometriomas had significantly higher levels of leptin in the PF than did patients with “deep” OEs [14]. Another report found that patients with PI at all stages of endometriosis showed higher PF leptin concentrations than patients with no implant, and the presence of OE had no significant main effect on leptin concentration [18]; however, isolated ovarian endometriosis is rare, as it is considered a marker for severe, deeply infiltrating endometriosis [22]. Furthermore, many endometriotic lesions, especially diaphragmatic and bowel lesions or atypical, non-pigmented PI, may not be visualized during surgery [23,24]. It is thus extremely difficult to exclude this variable.

Thus, peritoneal disease, but not ovarian endometriotic cysts, influences the concentration of leptin in PF in endometriosis; these two types of endometrial lesions may have different pathogenic mechanisms and distinct leptin-biosynthetic capacities [18]. Alternatively, the leptin may be sequestered into the cystic fluid of the OE [12]. We found increased levels of leptin in the EF compared to the PF of patients with both PI and OE; these variables were not correlated with each other. The increased levels of leptin in the EF may be the result of the slight decrease in leptin expression in ovarian tissue affected by endometrioma; this protein may have been secreted into the endometrioma and diffused in the chocolate fluid. In accordance with previous data, we believe that the concentration of leptin in the PF is influenced by PI; we also suggest that OEs influence leptin concentration in the EF.

Our findings show a strong positive correlation between the expression of leptin and OBR in OE and PI. A significant positive correlation was observed between leptin and OB-RL transcripts in ectopic endometria [25]. Although the difference was not statistically significant, previous data showed a modest positive correlation between the expression of leptin and that of OBR in patients with OEs [16]. Furthermore, these same authors demonstrated that leptin treatment induced OBR expression in endometriotic cells. We also demonstrated a significant positive correlation between PF leptin levels and the expression of leptin and OBR in PI, but this correlation was not observed in OE. In contrast, the expression of leptin and its receptor in OE correlated strongly and positively with leptin levels in EF. In contrast to our results, a significant negative correlation was observed between OBR transcripts and PF leptin levels in ectopic endometrium [25]. These significant positive correlations suggest that OBR may be induced in OE and PI by leptin levels in EF and PF, respectively.

Given the presence of large quantities of leptin in the OE, it remains unknown whether this inflammatory factor contributes to both the decreased oocyte reserve and the quality of the affected ovary. A prospective study revealed that elevated intra-ovarian leptin concentrations were associated with reduced ovarian stimulation and response, reduced follicle maturation, poorer embryo quality and a lower likelihood of successful pregnancy, suggesting that leptin modulates embryo quality and may serve as a sensitive marker of IVF outcomes [26]. We thus suggest that the increased leptin levels in the ovarian EF may play an important role in the reproductive abnormalities that accompany this disease, but further studies are required to support this hypothesis.

Disorders related to leptin deficit and leptin overabundance required the development of drugs that activate or inhibit the OBR [27]. The administration of the pegylated leptin peptide receptor antagonist (LPrA) or nonfunctional OBR (LeprdB) in a rat model of endometriosis demonstrated that disruption of leptin signaling can inhibits the establishment and development of endometriosis-like lesions that resemble peritoneal endometriotic foci [5]. A leptin mutant with antagonistic properties and other proteins that block leptin activity open up new possibilities for research and, eventually, therapy [28] for OE and similar diseases, which do not respond well to any available medication.

## Conclusion

In summary, this study shows that the expression of OBR is higher in the ovarian tissue affected by OE in infertile patients than in the normal ovarian tissue of fertile controls not affected by endometriosis. There was a positive and significant correlation between leptin and OBR expression in the OE and PI. We also demonstrated the presence of high levels of leptin in the chocolate fluid in the OE, wich correlated strongly and positively with the expression of leptin and OBR in the OE, while leptin levels in PF correlated with the expression of leptin and OBR in PI. These data suggest that leptin may have an important role in the physiopathology of OE through a modulatory interaction with its active receptor.

## Abbreviations

BMI: Body mass index; EF: Endometrioma fluid; OBR: Leptin receptor; OE: Ovarian endometrioma; PF: Peritoneal fluid; PI: Peritoneal implants.

## Competing interests

The authors declare they have no competing interests.

## Authors’ contributions

CR contributed to conception and design, acquisition, analysis and interpretation of data and drafting the article. HG and FC carried out the experiments described in the manuscripts. TP and AE performed clinical data collection. CR and MAO contributed to conception, analysis and interpretation of data and reviewed the article critically for important intellectual content. All authors read and approved the final manuscript.
